# New pathogenic variant in *DLX5*: New clues for a clinical spectrum from split-hand-foot malformation to fibular aplasia, tibial campomelia and oligosyndactyly

**DOI:** 10.3389/fgene.2023.1165780

**Published:** 2023-04-13

**Authors:** Anna Sifre-Ruiz, Amaia Sagasta, Erika Santos, Guiomar Perez de Nanclares, Karen E. Heath

**Affiliations:** ^1^ Pathology Service, Bioaraba Research Health Institute, Araba University Hospital, Vitoria-Gasteiz, Spain; ^2^ Radiodiagnostic Service, Araba University Hospital, Vitoria-Gasteiz, Spain; ^3^ Rare Diseases Research Group, Molecular (Epi)Genetics Laboratory, Bioaraba Research Health Institute, Araba University Hospital-Txagorritxu, Vitoria-Gasteiz, Araba, Spain; ^4^ Institute of Medical and Molecular Genetics (INGEMM), IdiPAZ, Hospital Universitario La Paz, Universidad Autónoma de Madrid, Madrid, Spain; ^5^ Skeletal Dysplasia Multidisciplinary Unit (UMDE), ERN-BOND, Hospital Universitario La Paz, Madrid, Spain; ^6^ CIBERER, ISCIII, Madrid, Spain

**Keywords:** DLX5, FATCO, fibular aplasia, tibial campomelia and oligosyndactyly, trio exome sequencing, split hand/foot malformation, SHFM, split hand/foot malformation with long bone deficiency, SHFLD

## Abstract

**Introduction:** FATCO (Fibular Aplasia, Tibial Campomelia and Oligosyndactyly) is a very infrequent skeletal dysplasia classified within the limb hypoplasia-reduction defects group whose genetic cause has not yet been identified. The advent of next-generation sequencing is enabling the diagnosis of diseases with no previously known genetic cause.

**Methods:** We performed a thorough autopsy on a fetus whose pregnancy was legally terminated due to severe malformations detected by ultrasound. A trio exome was run to identify the genetic cause and risk of recurrence. Previous literature of similar cases was systematically searched.

**Results:** Anatomopathological analyses revealed complete fibular aplasia, shortened and campomelic tibia, absent ankle joint, club right foot and a split foot malformation, leading to the diagnosis of FATCO. Exome sequencing showed that the female fetus carried a *de novo* nonsense variant in *DLX5*. The literature search permitted the collection of information on 43 patients with FATCO, the majority of whom were males diagnosed postnatally. In most cases, lower limbs were affected exclusively, but in 39.5% of cases the upper limbs were also affected.

**Conclusion:** The pathologies associated with *DLX5* variants encompass a wide spectrum of manifestations ranging from abnormalities exclusively in the hands and feet to long bones such as the tibia and fibula.

## 1 Introduction

In 1981, Hecht et al. reported for the first time the presence of bilateral foot defects, left fibular aplasia with homolateral tibial defects and associated bilateral hand defects in two-half siblings (a male and a female) from different fathers and a phenotypically normal mother (Hecht and Scott, 1981). It was not until 13 years later, in 1994, when similar findings were reported in a 24 weeks gestation male fetus. This is considered the first prenatal reporting (Capece et al., 1994). Later, the acronym “FATCO” (Fibular Aplasia, Tibial Campomelia and Oligosyndactyly) was proposed for this set of clinical features (Courtens et al., 2005). Since then, 43 cases of FATCO syndrome have been reported and no consistent genetic cause has been identified. The majority of the cases corresponded to male patients (63%), diagnosed during the postnatal period (58%). Although we lacked information regarding parental relationship on 17 (39%) of the cases, five cases (11.6%) were from consanguineous relationships. Details are provided in Supplementary Table S1.

As expected, every case presented the three major characteristics of FATCO syndrome (fibular aplasia, tibial campomelia and oligosyndactyly). Bilateral involvement of the lower limbs occurred in 12 (28%) of the cases. Although no significant laterality predominance was found, in the unilateral cases, the right lower limb was impaired in 17 (39.5%) of the cases while the left side involvement was reported in 14 (32.5%). Regarding upper limbs, only the hands were affected in 17 of the cases (39.5%); ten of them had bilateral involvement of both extremities, four had right hand defects and the other three had left hand deficiencies.

Regarding the presence of additional signs of the musculoskeletal system, six of the cases had other anomalies reported: two presented micrognathia, one of them in association with tracheo-oesophageal fistulae and oesophageal atresia (Courtens et al., 2005; Marinho et al., 2021); one had cleft palate and a left sided cleft lip (Kitaoka et al., 2009); one showed mild intercostal, subcostal retractions and brachycephaly (Georgeos and Elgzzar, 2022); one associated spina bifida occulta (Kavipurapu et al., 2021); and another one showed not specified minor facial anomalies (Nogueira et al., 2016).

Besides the musculoskeletal scheme, different alterations had been described in diverse systems (Supplementary Table S2). One subject was found to have severe hydrocephaly with associated haemorrhage of the lateral ventricles (Isik et al., 2019). There were three specimens showing cardiovascular alterations: one presented cardiomegaly with elevation of the apex (Hecht and Scott, 1981), and the other two with an interventricular septal defect (Bieganski et al., 2012; Igoche and Umaru, 2020); two cases had tracheo-oesophageal fistulae (one of them already mentioned above) (Nogueira et al., 2016; Marinho et al., 2021). One case had Klinefelter syndrome (Ekbote and Danda, 2012).

Familial history of FATCO cases was recorded in 30 patients (70%). Most of them (22 patients) did not show any remarkable familial history. Two cases associated with maternal familial history of skeletal anomalies (Supplementary Table S3): Patient #11’s mother showed bilateral partial skin syndactyly of toes II-III extending to the proximal interphalangeal joints (Bieganski et al., 2012); Patient #26’s mother showed syndactyly of the right hand and foot; his maternal grandmother presented right hand syndactyly and left hand oligodactyly, and died of unnatural unspecified causes and, finally, his maternal aunt had bilateral fibular aplasia (Guevara Zárate et al., 2018). The mother of another two cases presented with 20° thoracic scoliosis with convexity to the left side, but there was no clinical history of any skeletal dysplasia or malformation either in her or her family, even though both siblings from different fathers showed the FATCO syndromic complex (Hecht and Scott, 1981). Four cases were from parents who already had had a previous miscarriage or a clinical termination: a case of FATCO syndrome with brachycephaly in a couple who had a child with cerebral palsy of unknown cause and also had a termination due to multiple congenital anomalies (Georgeos and Elgzzar, 2022); a couple had a child with cerebral palsy, a child diagnosed with ADHD and a termination due to early Down syndrome diagnosis (Izadi and Salehnia, 2020); other two cases with the perinatal data briefly mentioned and termination reasons not described (Sezer et al., 2014; Hazan et al., 2016).

We describe a further case in whom a heterozygous *DLX5* pathogenic variant was detected.

## 2 Patient and methods

### 2.1 Case report

Our Pathology department received a 21+5 weeks gestational age female fetus referred due to right lower limb malformation in sonographic studies. The parents were a non-consanguineous couple. The father had no known health conditions. The mother was a 33-year-old woman who had Sjögren’s syndrome and stage 3 interstitial nephropathy associated with a diabetes insipidus. Her gestational history consisted in a previous early (7 weeks of gestation) abortion, and no deliveries. In this second pregnancy, the first trimester ultrasound detected a lack of movement in the right lower limb, associated with a permanent bent knee and an internal deviation of the middle segment. No drug or radiation exposure was reported.

A screening test, including an amniocentesis, was performed at 14+5 weeks of gestation, reporting low risk for the most common chromosomopathies (trisomy for chromosomes 13, 18, 21 and aneuploidies for sex chromosomes). The fetus showed a normal, 46, XX karyotype and no copy number variations were detected with a 44 k array-CGH (Agilent).

In the 21st week of gestation, a third echography confirmed the previous findings and the couple received specialized counselling on the treatment possibilities including orthopaedic surgery, amputation of the right lower limb or termination of the gestation. The couple decided to interrupt the current pregnancy and a fetal autopsy was performed.

### 2.2 Fetal autopsy

All procedures were performed in compliance with relevant legislation and institutional guidelines and were approved by the Ethics Committee of Araba University Hospital (2019-070).

The autopsy was carried out following our routine procedure based on the guidelines written by Linda M. Ernst, which includes photographs, measurements and radiological examination in addition to the external evaluation, before proceeding to dissection (Ernst, 2015). Macroscopic studies were followed and completed by histologic analyses of all organs including bones and articulations from upper and lower limbs.

### 2.3 Genetic tests

After obtaining written informed consent from the both parents, peripheral blood from the parents and fresh frozen muscle tissue from the fetus were collected, and genomic DNA was extracted. Whole exome sequencing (WES trio) was performed for the fetus and the parents. The Nextera DNA Flex Pre-Enrichment Library Prep and Illumina Exome Panel was used to capture the probe and conduct liquid hybridization with the genomic DNA library. The DNA fragments in the target region were enriched by WES analysis, and a whole exome library was established. Genomic libraries generated with S2/S4 Reagents Kits (Illumina) were sequenced on the NovaSeq6000 Sequencing System platform (Illumina). The median sequencing depth was 127.6X for the index and 144.6X and 158.5X for the parents.

The prioritisation and interpretation of variants have been carried out taking into account of clinical, genetic, scientific and population criteria, contrasting the data obtained with different populations, disease and gene databases (OMIM, gnomAD, ClinVar, ClinGen, HGMD Profesional) and using *in silico* pathogenicity prediction programs (SIFT, Polyphen, Mutation Taster, CADD V1.6). Subsequently, the variants were classified according to the American College of Medical Genetics and Genomics (ACMG) guidelines (Richards et al., 2015).

## 3 Results

### 3.1 Autopsy results

**FIGURE 1:**
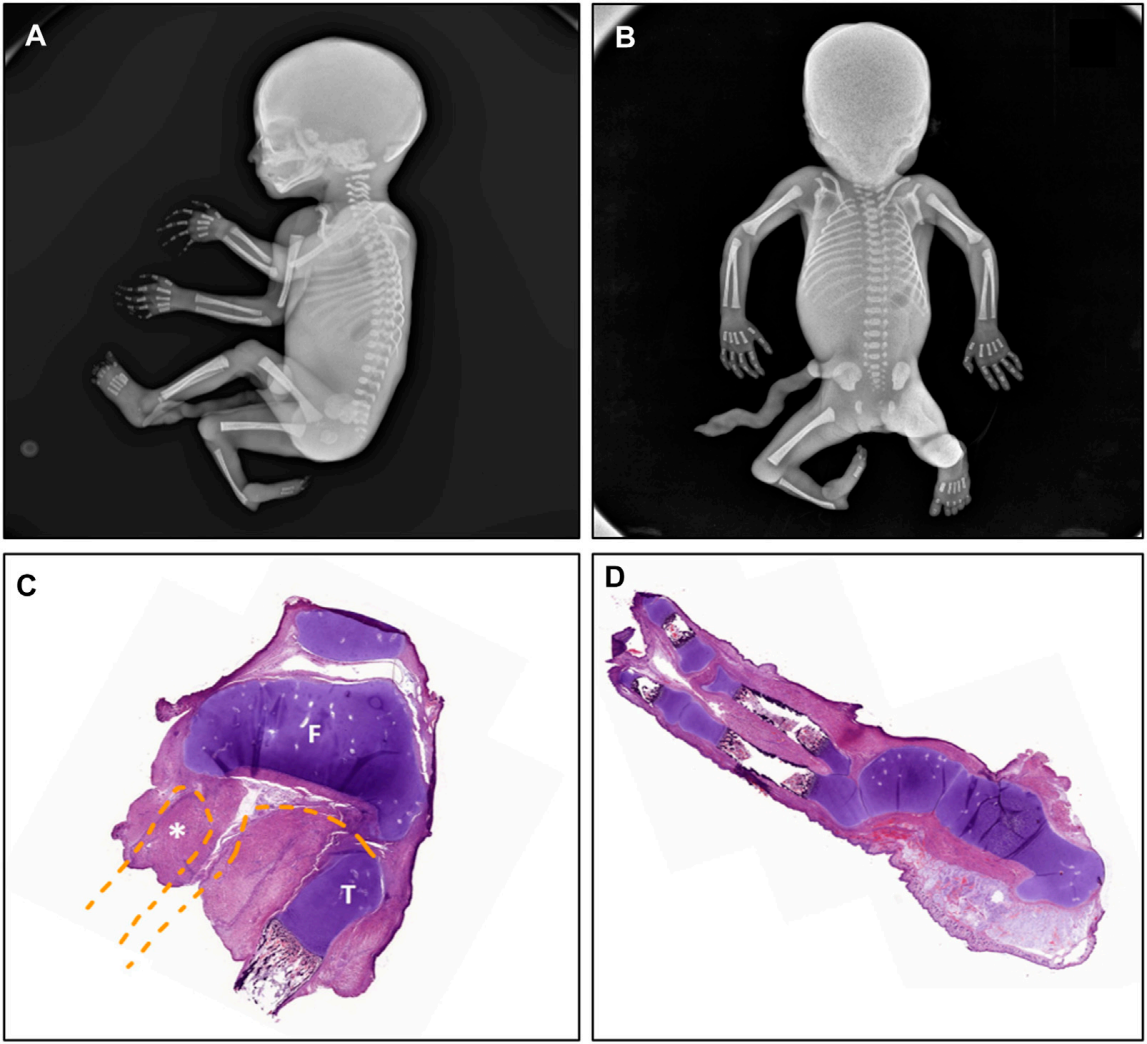
Radio-histopathological manifestations observed in the fetus compatible with FACTO syndrome. The radiologic study showed the presence of a right lower limb malformation with absence of long distal bone (**(A)**, lateral view) with aplasia of multiple metatarsal and tarsal foot bones, absence of the ankle joint and malrotation of the right foot (**(B)**, posterior view). The histopathological studies revealed **(C)** complete absence of the right fibula, partial proximal tibial hypoplasia with the presence of **(D)** vestigial tarsal bones, partial absence of metatarsal bones and oligodactyly (split foot malformation). F: femur; T: tibia; *: fibula.

Prior to starting the macroscopic evaluation of the specimen, two X-rays (antero-posterior and latero-lateral) were performed (Figures 1A, B). The fetus had a right lower limb malformation, consisting of a hyper-extended knee with internal deviation of the leg, a complete absence of a long distal bone (informed by the radiologist as “probably tibia”) and partial aplasia of some of the tarsal bones of the right foot with mal-rotation of the mentioned foot. No other skeletal abnormalities were detected in X-rays analyses.

Anteroposterior, lateral and close-up photographs were taken. Externally, the lower limbs were asymmetrical, the right limb was shorter and the knee was bent.

When the limb was dissected, complete fibular aplasia was observed (stage II of Achterman and Kalamchi) (Achterman and Kalamchi, 1979). The tibia was shortened (measurements: right: 2.8 cm; left: 4.4 cm) and campomelic (anterior bowing of the two inferior thirds of the bone). The ankle joint was absent. The right foot showed a club and split malformation, which consisted in vestigial tarsal bones (partial absence of metatarsal bones) and oligodactyly (only two rays were found).

The histopathological examination of the knee revealed that the preserved long bone articulated with the femur, therefore the fetus had an absent fibula (Figure 1C) and confirmed the foot alterations (Figure 1D). Developmental state appeared concordant to the gestational age and ossification nuclei seemed correctly formed. No other visceral nor systemic anomalies were noted, either externally, internally or during the histological examination.

### 3.2 Genetic results

Exome trio sequencing revealed a heterozygous nonsense variant, NM_005221.5:c.219C>G p.(Tyr73*) in *DLX5* in the fetus (sequencing depth 236X), which was absent in parental DNA (raw data are available at the European Nucleotide Archive, submission number ERP144873). This variant is absent from the gnomAD population database (https://gnomad.broadinstitute.org/) and it is predicted to result in the premature truncation of the protein. The variant was classified as pathogenic (PVS1_moderate, PS2, PM2) using the American College of Medical Geneticists (ACMG) variant classification guidelines (Richards et al., 2015).

## 4 Discussion

FATCO syndrome forms part of the limb hypoplasia-reduction defects group of the Nosology of genetic skeletal disorders (Unger et al., 2023), along with 41 other dysplasias, including Fanconi anemia (MIM 607139), Holt-Oram syndrome (MIM 142900), Split-hand-foot malformation with long bone deficiency (SHFLD, MIM 119100), Furhmann syndrome (MIM 228930) and Al-Awadi Raas-Rothschild limb-pelvis hypoplasia-aplasia (MIM 276820).

FATCO is an uncommon entity whose prevalence has been reported to be less than one per million births (https://www.orpha.net/consor/cgi-bin/OC_Exp.php?lng=ES&Expert=2,492). The majority of reported cases, as with the case reported here, present with the defined criteria for the complex, i.e., Fibular Aplasia, Tibial Campomelia and Oligosyndactyly (Hecht and Scott, 1981; Cuillier et al., 2004; Courtens et al., 2005; Monteagudo et al., 2006; Kitaoka et al., 2009; Karaman and Kahveci, 2010; Vyskocil et al., 2011; Bieganski et al., 2012; Ekbote and Danda, 2012; Goyal, 2014; Sezer et al., 2014; Bastaki et al., 2015; Hazan et al., 2016; Nogueira et al., 2016; Smets et al., 2016; D’Amato Gutiérrez and Palacio Díaz, 2016; Abdalla and El-Beheiry, 2017; Ahmad et al., 2017; Petricevic, 2017; Guevara Zárate et al., 2018; Otaryan et al., 2018; Isik et al., 2019; Önder Yılmaz et al., 2019; Igoche and Umaru, 2020; Izadi and Salehnia, 2020; Kavipurapu et al., 2021; Marinho et al., 2021; Mishra and Verma, 2021; Yucel Celik et al., 2021; Mumtaz Hashmi et al., 2022; Georgeos and Elgzzar, 2022; Georgescu et al., 2022), but other systemic manifestations have been described in some cases (Hecht and Scott, 1981; Courtens et al., 2005; Kitaoka et al., 2009; Bieganski et al., 2012; Nogueira et al., 2016; Isik et al., 2019; Igoche and Umaru, 2020; Kavipurapu et al., 2021; Marinho et al., 2021; Georgeos and Elgzzar, 2022). Although data is scarce, no significant difference in laterality nor with the frequency of other extra-skeletal conditions has been found, probably due to the rarity of this entity.

To the present day, the causality of FATCO is yet to be identified. A genetic basis for this condition remains unknown and its inheritance model is still unclear; sporadic occurrence (due to postzygotic genetic alterations) has been proposed (Lewin and Opitz, 1986), but also recessive inheritance or gonadal mosaicism for a dominant mutation (Hecht and Scott, 1981). Historically, the majority of genetic studies performed were chromosomal studies, including karyotypes and CGH arrays. As no chromosomal alteration was identified, candidate genes were sequenced. These included those which participate in skeletal embryogenesis and the maintenance of bone and cartilage, such as NOTCH, WNT, TGFβ and BMP signaling pathways, that cause other limb hypoplasia-reduction defects or ectrodactyly (Guasto and Cormier-Daire, 2021; Vlashi et al., 2022)). Various studies have excluded *WNT7A,* which is responsible for Furhmann syndrome (Kitaoka et al., 2009; Karaman and Kahveci, 2010; Petricevic, 2017; Önder Yılmaz et al., 2019; Yucel Celik et al., 2021) and *WNT10B* and *P63* were also excluded as causative genes (Bieganski et al., 2012).

Here, exome trio sequencing revealed the presence of a heterozygous *de novo* pathogenic *DLX5* (distal-less homeobox gene 5) variant, p.(Tyr73*) in the fetus. The prematurely truncated mutant protein is predicted to lack the homeobox domain (amino acids 137–196), important in DNA binding and protein interactions.

*DLX5* is considered to play an important role in early limb morphogenesis (Sowińska-Seidler et al., 2014; Marques et al., 2016). The Distal-less (Dll) gene of *Drosophila* encodes a homeodomain protein that is one of the first to be expressed during leg primordia and cephalic development (Cohen et al., 1989; O’Hara et al., 1993; Panganiban and Rubenstein, 2002). Adult Dll *Drosophila* mutants present reduction and dysmorphogenesis of distal segments of most appendages, including legs, antennae, and mouth parts, indicating that the Dll activity is required for appendage proximo-distal (PD) organization during early larval stages (Cohen et al., 1989; Cohen, 1990; Gorfinkiel et al., 1999). In vertebrates, six Dlx genes linked in three bigenic pairs, in a tandem convergent manner are present: Dlx1/Dlx2; Dlx3/Dlx4 and Dlx5/Dlx6 (Panganiban and Rubenstein, 2002). The three Dlx bigenic clusters are located in the same chromosomes where the HOX complexes are found, also implicated in various developmental pathways. For example, in humans, *DLX5* and *DLX6* are linked to the HOXA cluster on chromosome 7 (Simeone et al., 1994).

Dlx5/6 double mutant mice present a limb malformation deriving from an early apical ectodermal ridge defect (Merlo et al., 2002; Robledo et al., 2002; Conte et al., 2016). The functional characterization of the DLX5/6 regulatory region identified 26 tissue-specific enhancers capable of directing the expression of Dlx5/6 either in the limb, in craniofacial regions, and/or in the brain during development (Scherer et al., 1994). The limb and facial malformations observed in Dlx5/6 double mutant mice are similar to those observed in split hand foot malformation type I (SHFM1, MIM 183600), a clinically and genetically heterogeneous human ectrodactyly. Although, SHFM1 patients show mostly autosomal dominant inheritance with variable expressivity and reduced penetrance, in the Dlx5/6 double mutant model, the ectrodactyly phenotype is mostly recessive with rare defects observed in heterozygous mice (Robledo et al., 2002; Conte et al., 2016).

In humans, Split-hand/foot malformation (SHFM1, MIM 183600) has been shown to be caused by various genetic alterations involving *DLX5*: chromosomal aberrations involving the chromosomal region 7q21.3 (reviewed by (Rasmussen et al., 2016)), pathogenic variants in *DLX5* (Shamseldin et al., 2012; Sowińska-Seidler et al., 2014; Wang et al., 2014), or dysregulation of *DLX5/DLX6* expression by long-range position effects of one or more of the 26 tissue-specific enhancers (Birnbaum et al., 2012). SHFM1 can be isolated or syndromic with incomplete penetrance and a highly variable clinical expression within and between families, and sex-related segregation distortion (Sowińska-Seidler et al., 2014). It is characterized by a reduction in the number of digits and an augmented separation between the anterior and posterior rays.

Although classified in different nosology groups (Unger et al., 2023), phenotypic overlap of SHFM forms and FATCO have led to the suggestion of similar genetic background (Bieganski et al., 2012), however, few molecular studies have been undertaken. The molecularly confirmed case described here validates this hypothesis.

But, the scientific dilemma is what causes the differences in clinical phenotype between the truncating *DLX5* variants identified in patients with SHFM1 and the truncating variant observed in this proband with FATCO syndrome? The location of the variants in the protein structure does not appear to play a role as the nonsense variants reported to date all lack the homeobox domain (Sowińska-Seidler et al., 2014). Thus, at present, one can only hypothesize that spatial and temporal regulation may influence the clinical phenotype. With the ever increasing use of genome sequencing, further cases may reveal the pathogenetic mechanism for the clinical heterogeneity.

## Data Availability

The datasets presented in this study can be found in online repositories. The names of the repository/repositories and accession number(s) can be found below: https://www.ebi.ac.uk/ena, ERP144873.
