# Supplementary material for: New pathogenic variant in DLX5: New clues for a clinical spectrum from split-hand-foot malformation to fibular aplasia, tibial campomelia and oligosyndactyly
Source: Front Genet. 2023 Apr 13;14:1165780. doi: 10.3389/fgene.2023.1165780 (PMC10133553; doi:10.3389/fgene.2023.1165780)
Supplement: Supplementary file 2 [file Table1.DOCX]

Supplementary Material

# Supplementary Tables

Submitted as Excel files

## Supplementary Table 1: Clinical characteristics of the patients selected after the literature search. The age at diagnosis, sex of the patient, skeletal alterations present in both upper and lower extremities and the genetic studies performed are described. F: female; M: male; ND: non-disponsable; WoG: weeks of gestation; aCGH: comparative genomic hybridization array;

## Supplementary Table 2: Clinical manifestations not related to the defining criteria of FATCO presented by patients. Upper limb abnormalities are also excluded.

## Supplementary Table 3: Familial history reported in the FATCO cases of the present series. Both skeletal alterations and other described manifestations are included. ND: non-disponsable.

# Reference list:

Abdalla, E. M., and El-Beheiry, A. A. (2017). Overlap between Fibular Aplasia, Tibial Campomelia, and Oligosyndactyly and Fuhrmann’s Syndromes in an Egyptian Female Infant. *J. Pediatr. Genet.* 6, 118–121. doi: 10.1055/s-0036-1597931.

Ahmad, K., Ahmad Malla, H., and Dawood, S. (2017). FATCO Syndrome (Fibular Aplasia, Tibial Campomelia, Oligosyndactyly with Talar Aplasia). A Case Study. *Ortop. Traumatol. Rehabil.* 19, 75–78. doi: 10.5604/15093492.1235280.

Bastaki, L. A., Al-Hathal, M., Sadik, D. I., Alrohaif, H. E., Yousef, H. Y., and Khallaf, M. G. (2015). A case report of FATCO syndrome. Middle East J. Med. Genet. 4, 28–30. doi: 10.1097/01.MXE.0000456627.22542.40.

Bieganski, T., Jamsheer, A., Sowinska, A., Baranska, D., Niedzielski, K., Kozlowski, K., et al. (2012). Three new patients with FATCO: Fibular agenesis with ectrodactyly. Am. J. Med. Genet. Part A 158A, 1542–1550. doi: 10.1002/ajmg.a.35369.

Courtens, W., Jespers, A., Harrewijn, I., Puylaert, D., and Vanhoenacker, F. (2005). Fibular aplasia, tibial campomelia, and oligosyndactyly in a male newborn infant: A case report and review of the literature. Am. J. Med. Genet. Part A 134A, 321–325. doi: 10.1002/ajmg.a.30441.

Cuillier, F., Cartault, F., and Lemaire, P. (2004). Absence of fibula type II. TheFetus.net. Available at: https://thefetus.net/content/absence-of-fibula-type-ii/ [Accessed September 20, 2022].

D’Amato Gutiérrez, M., and Palacio Díaz, F. A. (2016). [A case report of a patient with FATCO syndrome: fibular aplasia, tibial campomelia and oligosyndactyly]. Arch. Argent. Pediatr. 114, e167-70. doi: 10.5546/aap.2016.e167.

Ekbote, A. V, and Danda, S. (2012). A case report of fibular aplasia, tibial campomelia, and oligosyndactyly (FATCO) syndrome associated with Klinefelter syndrome and review of the literature. *Foot Ankle Spec.* 5, 37–40. doi: 10.1177/1938640011422594.

Georgeos, M. K., and Elgzzar, D. R. (2022). Newborn Male With Fibular Aplasia, Tibial Campomelia, and Oligosyndactyly Syndrome: A New Case Report Putting the Condition Under Spotlight. Cureus 14, e21702–e21702. doi: 10.7759/cureus.21702.

Georgescu, T., Ionescu, O., Toader, O. D., Bacalbasa, N., and Pop, L. G. (2022). Fibular hemimelia. J. Med. Life 15, 587–588. doi: 10.25122/jml-2021-0397.

Goyal, N. (2014). FATCO Syndrome Variant - Fibular Hypoplasia, Tibial Campomelia and Oligosyndactyly –– A Case Report. J. Clin. DIAGNOSTIC Res. 8, LD01–LD02. doi: 10.7860/JCDR/2014/9275.4787.

Guevara Zárate, J. M., Rodríguez, A. J., and Ortiz, C. A. (2018). Síndrome de FATCO (aplasia fibular, campomelia de tibia y oligosindactilia) en paciente masculino: reporte de caso. Rev. Colomb. Médicina Física y Rehabil. 28, 70–74. doi: 10.28957/rcmfr.v28n1a7.

Hazan, F., Katipoğlu, N., Kaya Kılıç, F., Hekimoğlu, Ü., Olukman, Ö., Çalkavur, Ş., et al. (2016). FATCO syndrome: a new case and review of the literature. Pamukkale Med. J. 9, 236–239. doi: 10.5505/ptd.2016.10327.

Hecht, J. T., and Scott, C. I. (1981). Limb deficiency syndrome in half-sibs. Clin. Genet. 20, 432–437. doi: https://doi.org/10.1111/j.1399-0004.1981.tb01054.x.

Igoche, D. P., and Umaru, H. (2020). Fibular Aplasia ‑ Tibial Campomelia ‑ Oligodactyly Syndrome Phenotype with Isolated Ventricular Septal Defect in an African Child. *J. Pract. Cardiovasc. Sci.* 6, 68–70. doi: 10.4103/jpcs.jpcs.

Isik, E., Atik, T., and Ozkinay, F. (2019). The first report of fibular agenesis, tibial campomelia, and oligosyndactyly syndrome with hydrocephaly. Clin. Dysmorphol. 28, 38–40. doi: 10.1097/MCD.0000000000000247.

Izadi, M., and Salehnia, N. (2020). Prenatal Diagnosis of FATCO Syndrome (Fibular Aplasia, Tibial Campomelia, and Oligosyndactyly) with 2D/3D Ultrasonography. Ultrasound Int. Open 06, E44–E47. doi: 10.1055/a-1225-4388.

Karaman, A., and Kahveci, H. (2010). A male newborn infant with fatco syndrome (fibular aplasia, tibial campomelia and oligodactyly): a case report. *Genet. Couns.* 21, 285–8. Available at: http://ovidsp.ovid.com/ovidweb.cgi?T=JS&PAGE=reference&D=emed11&NEWS=N&AN=359730161.

Kavipurapu, S. P., Maganthi, M., Sundar, L. S., and Ramya, S. (2021). Fibular Aplasia, Tibial Campomelia, Oligo-Syndactyly Syndrome and Probable Femur Fibula Ulna Syndrome- Case Reports. J. Clin. DIAGNOSTIC Res. 15, SD01–SD03. doi: 10.7860/JCDR/2021/46108.14551.

Kitaoka, T., Namba, N., Kim, J. Y., Kubota, T., Miura, K., Miyoshi, Y., et al. (2009). A Japanese Male Patient with Fibular Aplasia, Tibial Campomelia and Oligodactyly’: An Additional Case Report. Clin. Pediatr. Endocrinol. 18, 81–86. doi: 10.1297/cpe.18.81.

Matalon, D. R., Bhoj, E. J., Li, D., McDougall, C., Schindewolf, E., Khalek, N., et al. (2023). Genomic sequencing in a cohort of individuals with fibular aplasia, tibial campomelia, and oligosyndactyly (FATCO) syndrome. Am. J. Med. Genet. A. 191, 977–982. doi: 10.1002/ajmg.a.63105.

Marinho, M., Nunes, S., Lourenço, C., Melo, M., Godinho, C., and Nogueira, R. (2021). Prenatal diagnosis of fibular aplasia-tibial campomelia-oligosyndactyly syndrome: Two case reports and review of the literature. J. Clin. Ultrasound 49, 625–629. doi: 10.1002/jcu.22969.

Mishra, P. K., and Verma, M. (2021). Fibular Hypoplasia, Talar Aplasia, Absent Proximal Tibial Growth Plate and Oligosyndactyly (Variant of Fibular Aplasia, Tibial Campomelia, and Oligosyndactyly Syndrome) – Paucity of Case Reports with Evolving Definition. J. Orthop. CASE REPORTS 11, 46–49. doi: 10.13107/jocr.2021.v11.i08.2360.

Monteagudo, A., Dong, R., and Timor-Tritsch, I. E. (2006). Fetal Fibular Hemimelia. J. Ultrasound Med. 25, 533–537. doi: 10.7863/jum.2006.25.4.533.

Mumtaz Hashmi, H., Shamim, N., Kumar, V., Mirza, A., Kirmani, S., Irfan, B., et al. (2022). A case report on Fibular Aplasia, Tibial Campomelia, Oligosyndactyly Syndrome variant in a Male Infant. J. Pak. Med. Assoc. 72, 975–977. doi: 10.47391/JPMA.3793.

Nogueira, R., Sá, J., Varela, C., Amorim, G., Valente, F., and Tavares, P. (2016). Four FATCO syndrome cases: clinical, autopsy and placental features with literature review update. J. Med. Biomed. Appl. Sci. 4, 20–25.

Önder Yılmaz, H., Topak, D., Yılmaz, O., and Çakmaklı, S. (2019). A Turkish Female Twin Sister Patient with Fibular Aplasia, Congenital Tibia Pseudoarthrosis, Oligosyndactyly, and Negative WNT7A Gene Mutation. *J. Pediatr. Genet.* 8, 95–99. doi: 10.1055/s-0038-1675837.

Otaryan, K. K., Sakvarelidze, N. Y., and Gagaev, C. G. et all (2018). FATCO syndrome: a case of prenatal diagnosis in the second trimester of pregnancy. *Prenat. Diagn.* doi: 10.21516/2413-1458-2018-17-4-355-359.

Petricevic, J. (2017). First Case of Bilateral Fibular Aplasia, Tibial Campomelia and Oligodactyly Syndrome (FATCO Syndrome). Clin. Stud. Med. Case Reports 4, 1–3. doi: 10.24966/CSMC-8801/100046.

Sezer, O., Gebesoglu, I., Yuan, B., Karaca, E., Gokce, E., and Gunes, S. (2014). Fibular aplasia, tibial campomelia, and oligosyndactyly: a further patient with a 2-year follow-up. Clin. Dysmorphol. 23, 121–126. doi: 10.1097/MCD.0000000000000051.

Smets, G., Vankan, Y., and Demeyere, A. (2016). A Female Newborn Infant with FATCO Syndrome Variant (Fibular Hypoplasia, Tibial Campomelia, Oligosyndactyly) - A Case Report. J. Belgian Soc. Radiol. 100, 41. doi: 10.5334/jbr-btr.929.

Vyskocil, V., Dortova, E., Dort, J., and Chudacek, Z. (2011). FATCO syndrome - fibular aplasia, tibial campomelia and oligosyndactyly. Jt. Bone Spine 78, 217–218. doi: 10.1016/j.jbspin.2010.08.013.

Yucel Celik, O., Gultekin Calik, M., Keles, A., Tos, T., Yucel, A., and Sahin, D. (2021). Prenatal differential diagnosis of fibular agenesis, tibial campomelia and oligosyndactyly. Clin. Dysmorphol. 30, 147–149. doi: 10.1097/MCD.0000000000000366.
